# PPARs in Clinical Experimental Medicine after 35 Years of Worldwide Scientific Investigations and Medical Experiments

**DOI:** 10.3390/biom14070786

**Published:** 2024-07-01

**Authors:** Anna Skoczyńska, Monika Ołdakowska, Agnieszka Dobosz, Rajmund Adamiec, Sofya Gritskevich, Anna Jonkisz, Arleta Lebioda, Joanna Adamiec-Mroczek, Małgorzata Małodobra-Mazur, Tadeusz Dobosz

**Affiliations:** 1 Department of Internal and Occupational Medicine and Hypertension, Wroclaw Medical University, Borowska 213, 50-556 Wroclaw, Poland; anna.skoczynska@umw.edu.pl; 2 Department of Forensic Medicine, Division of Molecular Techniques, Wroclaw Medical University, M. Sklodowskiej-Curie 52, 50-369 Wroclaw, Poland; monika.oldakowska@umw.edu.pl (M.O.); anna.jonkisz@umw.edu.pl (A.J.); arleta.lebioda@umw.edu.pl (A.L.); malgorzata.malodobra-mazur@umw.edu.pl (M.M.-M.); tadeusz.dobosz@umw.edu.pl (T.D.); 3 Department of Basic Medical Sciences and Immunology, Division of Basic Medical Sciences, Wroclaw Medical University, Borowska 211, 50-556 Wrocław, Poland; 4 Department of Diabetology and Internal Medicine, Wroclaw Medical University, Borowska 213, 50-556 Wroclaw, Poland; rajmund.adamiec@umw.edu.pl; 5 Department of Internal Medicine, Faculty of Medical and Technical Sciences, Karkonosze University of Applied Sciences, Lwówiecka 18, 58-506 Jelenia Góra, Poland; 6 Department of Ophthalmology, Wroclaw Medical University, Borowska 213, 50-556 Wroclaw, Poland; joanna.adamiec-mroczek@umw.edu.pl

**Keywords:** PPARs, peroxisome proliferator-activated receptor, clinical application, experimental therapy

## Abstract

This year marks the 35th anniversary of Professor Walter Wahli’s discovery of the PPARs (Peroxisome Proliferator-Activated Receptors) family of nuclear hormone receptors. To mark the occasion, the editors of the scientific periodical Biomolecules decided to publish a special issue in his honor. This paper summarizes what is known about PPARs and shows how trends have changed and how research on PPARs has evolved. The article also highlights the importance of PPARs and what role they play in various diseases and ailments. The paper is in a mixed form; essentially it is a review article, but it has been enriched with the results of our experiments. The selection of works was subjective, as there are more than 200,000 publications in the PubMed database alone. First, all papers done on an animal model were discarded at the outset. What remained was still far too large to describe directly. Therefore, only papers that were outstanding, groundbreaking, or simply interesting were described and briefly commented on.

## 1. Introduction

It is widely believed that the Peroxisome Proliferator-Activated Receptor (PPAR) was described around 1974 by Prof. Walter Wahli. Unfortunately, among his 350 or so published papers, the fundamental, foundational, and pioneering work could not be found (and as there are at least two other contenders for priority [1,2]), we asked Prof. Walter Wahli for a statement on this matter. In response, Prof. W. Wahli identified one of his papers as the first (starting) paper on this topic, indirectly confirming his priority and providing its biographical data [3]. The paper cited is from March 1992 and concerns not a human but the amphibian *Xenopus laevis*, which, incidentally, explains our difficulty in finding it, since PubMed lists, at the time of writing these words, over 300,000 abstracts and full-text papers dealing with PPARs.

**Figure 1:**
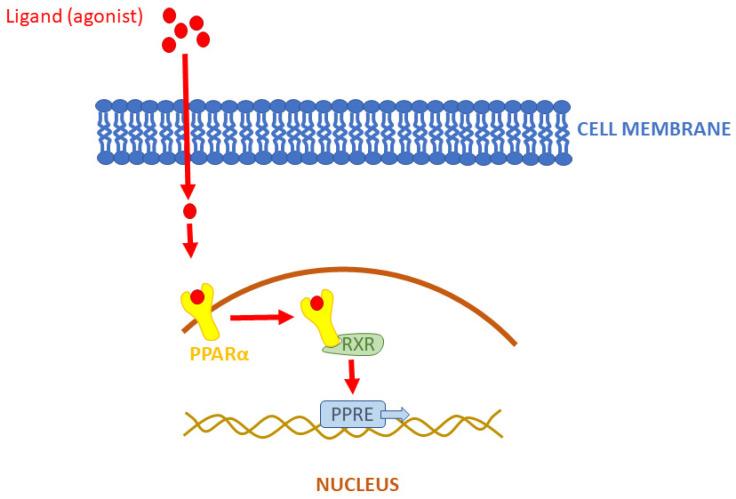
Pattern of PPARα activity. RXR—retinoid X receptor, PPRE—gene promoter.

To finish this review on time, despite the challenging work of all authors, almost all work done on animal models (including *Xenopus*) was discarded at the outset, as it was assumed that they are usually a preliminary step before work on human material, as animal models are generally very different from humans [4]. Notably, the ‘PPARs fad’ is slowly passing relative to the publication peak of 2011–2013, when there were 5–6 new publications daily. In the last few years, it has been 4-3 papers per day, following a decreasing trend. New papers are located on the right, descending branch of the Gauss curve. Assuming its complete symmetry, the last single documents in this field are expected around 2040. Tahri-Joutey et al. [5] report (following [6]) that PPAR-alpha, the first of the known receptors, arose at the time of the evolutionary breakthrough of fish and mammals, about 200 million years ago, and evolved three times faster than the other receptors beta/delta and gamma. In 2006, a concise but comprehensive and complete description of the mechanism of action of PPARs appeared in print with co-authorship by Lathion [7]. The paper explained the mechanism of action using PPAR-alpha as an example; however, the activation of all PPAR is very similar. The authors describe the following sequence of events: first, a PPAR heterodimer is formed with the retinoid X receptor (RXR), and then the newly formed complex binds a ligand that defines the end effector. The ligand in question (a substance from a long list of natural or artificial compounds) can be an agonist (increasing activity) or an antagonist (decreasing activity). The described complex (PPAR plus RXR plus ligand) binds to a specific DNA sequence in the gene promoter (PPRE) and triggers transcription (Figure 1).

**Figure 2:**
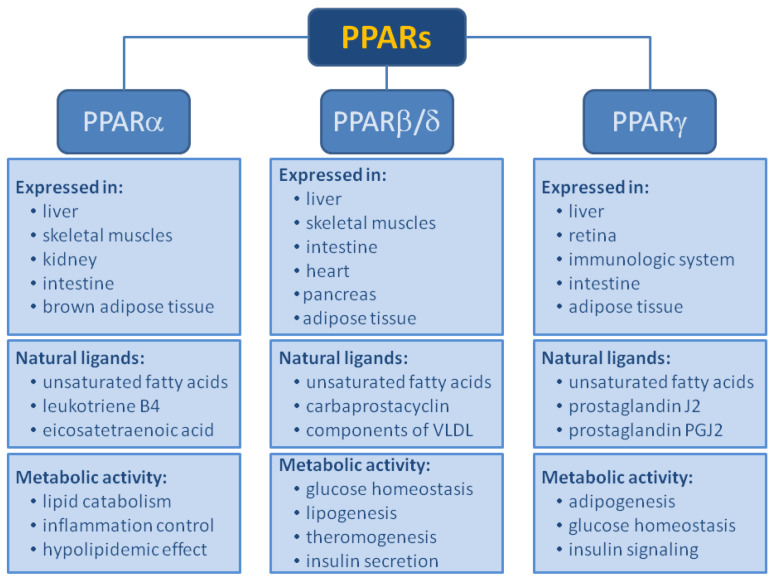
The characteristic of the three isoforms of PPARs: PPARα, PPARβ/δ, and PPARγ (expression, function, and the list of main ligands activating the particular peroxisome proliferator).

Figure 2 shows basic information about PPAR isoforms. However, it should be noted that the number of PPAR types is not fully established. For the first few years, it was thought that there were four [3]. Then, their number was reduced to three, but later molecular studies show that there are four (named in 1992: alpha, beta, gamma, and delta) [8].

### 1.1. Peroxisome Proliferator-Activated Receptor Alpha (PPARα)

PPARα is a ligand-activated transcriptional factor that belongs to the nuclear receptor superfamily. It is also known as N1C1 (nuclear receptor subfamily 1, group C, member 1). PPARα is strongly expressed in tissues with elevated fatty acid catabolism, such as the liver, heart, skeletal muscles, brown adipose tissue, and intestine. It regulates the transcription of multiple genes involved in the intracellular metabolism of lipids and is a major regulator of fatty acid and triglyceride homeostasis [9,10,11]. PPAR-alpha performs the direct transcriptional control of genes implicated in mitochondrial and peroxisomal β-oxidation and microsomal ω-oxidation also. Modulating the activities of all three fatty acid oxidation systems plays a crucial role in energy expenditure. It also regulates the expression of proteins involved in the transport and β-oxidation of free fatty acids (FFAs) and lipoprotein metabolism genes [12].

The human PPAR-alpha receptor gene (*PPARA*) is located on chromosome 22 at the position *22q12-q13.1*. PPAR-alpha receptor shares a common characteristic with the other members of the PPAR family (PPAR-beta/delta and PPAR-gamma) and has five domains [13]. Among these are the ligand-binding domain, dimerization domain (a dimer forms during the reaction with the PPAR-responsive element), and DNA-binding domain, which binds to the promoter of different genes via the zinc finger. There are two stages in PPAR-alpha transcriptional activation. In the first stage, PPAR-alpha is activated by the natural ligand or agonist, and the second stage is started by the formation of heterodimer PPAR-alpha with a retinoid X receptor (RXR). Then the PPAR-alpha-RXR complex recognizes many genes, binding to PPRE via the zinc finger of the DNA-binding domain. The PPRE is a sequence of nucleotides: AGGTCA, AGGTCA. This sequence is always the same on every gene to which the PPAR-alpha-RXR complex binds [14]. For example, there may be up to 80 different genes regulated by PPAR-alpha in hepatocytes. This action includes the stimulation of the apolipoprotein AI genes, the apolipoprotein AII genes, and the lipoprotein lipase (LPL) gene, and the inhibition of apolipoprotein CIII [14].

### 1.2. Peroxisome Proliferator-Activated Receptor Beta/Delta (PPARβ/δ)

This receptor type, perhaps because it has numerous links to tumorigenesis, is quite popular; there are about 1200 abstracts and full papers on this topic in the PubMed database. In the late 1980s, it was widely believed that there were two separate receptors, PPAR-beta and PPAR-delta. However, closer examination showed that they were the same, so some researchers felt it was necessary to eliminate one of them. Unfortunately, a big mess in naming ensued, and from Google data, it can be calculated that as many as 83% of researchers use the name “beta”, 13% use the name “delta”, and 4% refuse to declare their choice and use both names, e.g., “beta/delta”.

### 1.3. Peroxisome Proliferator-Activated Receptor Gamma (PPARγ)

Cardiovascular diseases such as diabetes, hypertension, obesity, and dyslipidemia are the most common causes of death and severe disability. Their treatment consists primarily of modifying environmental factors to normalize metabolic changes, but genetic information is also essential in developing these diseases. The primary gene whose mutation can cause multistep disorders leading to the development of metabolic syndrome and atherosclerosis is the peroxisome proliferator-activated receptor gene γ (*PPARG*). This gene is located on chromosome *3p25*. Its expression can start from different promoters, depending on the site of origin, which results in the possibility of 3 isoforms of this receptor protein: PPARγ-1 (adipose tissue, muscle, heart, liver), PPARγ-2 (mainly adipose tissue), and PPARγ-3 (adipose tissue and colon). The expression of the PPARγ gene depends on, among other things, insulin and food availability, especially free fatty acids. PPARγ modulates insulin sensitivity and immune system activity by controlling the transcription of many proteins essential for carbohydrate and lipid metabolism. PPAR-gamma agonists in type 2 diabetes resulted in lower serum levels of pro-inflammatory markers, such as CRP, white blood cells, soluble CD40, MMP-9, amyloid A, and TNFα [15].

### 1.4. Pan-PPAR (Alpha, Beta/Delta, Gamma)

The phenomenon of the combined treatment of several seemingly separate (if only partially) phenomena bears the prefix “pan-” in the case of the receptors described; it is about pan-ligands (usually agonists) and pan-PPARs. This phenomenon has been discovered and described as many as three times, yet it is little known and rarely used. The term “pan-PPAR” was noted for the first time in a paper by Cullingford et al. from 2002 [16]. It appeared for the second time in the literature reviewed in a 2007 paper by Rudolph et al. [17]. The authors treated all three PPAR isoforms (alpha, beta/delta, and gamma) together, indirect evidence of which is the new term “pan agonist”. Notwithstanding the above, three years later, Huang et al. [18] stated for the third time that separate testing of individual alpha, beta/delta, and gamma activities is incorrect, as the sum of the activities of all three isoforms should be considered. As an argument, they cited that using one of the experimental ligands initially significantly reduces, by decreasing the PPARα activity, the rate of cancer cell proliferation. Still, the subsequent increase in activity within the pan-PPAR phenomenon, specifically PPAR-gamma activity, spoils the whole effect (Table 1).

## 2. Aim of the Work

Of particular note is that about 300,000 papers have been published on PPARs. This is undoubtedly an important topic that should be studied in depth. The published works go in different directions in learning about PPARs and sometimes the research results duplicate each other, thus supporting the validity and correctness of the results obtained by other research teams. This article was written to systematize and collect the existing knowledge about PPARs. It used the following databases: PubMed, EBSCO, Scopus, OMIM, and Google Scholar. By demonstrating the current state of knowledge, it is hoped that this work will contribute to better research planning, reduce unnecessary repetition, accelerate the growth of knowledge, and produce more interesting results.

## 3. Results

### 3.1. Introduction

The avalanche of papers dealing with PPARs moved very slowly. Quoting from Vamecq et al. [19], possibly the first pioneering paper on PPARs appeared in the second part of 1965, and from the beginning of 1966 until the end of 1991, on average, a new paper appeared every few months. For example, in 1988, two papers by Reddy et al. [20,21] appeared, writing about PPARs in liver cancer. Another author wrote about a “steroid...hormone receptor” the same year [2]. In contrast, Prof. W. Wahli gave us the date 1992 as the beginning of research on PPARs [8]. Independently, we found the work of Wahli and Martinez in 1991, in which the authors wrote about PPAR and its positive and negative regulations [22].

**Figure 3:**
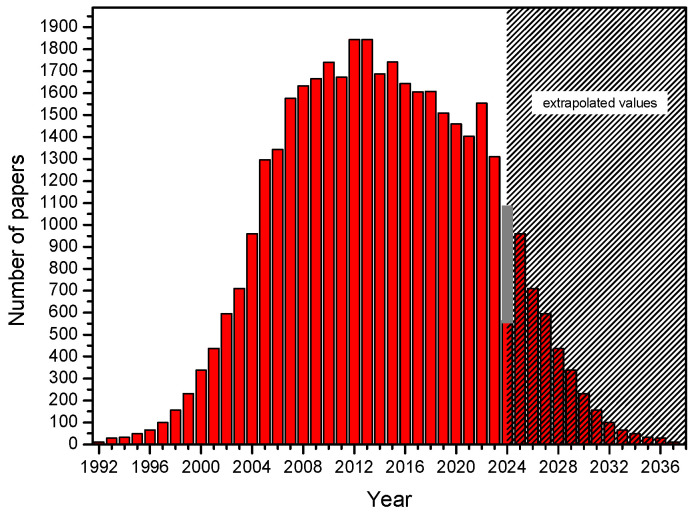
Number of papers published in 1992–2024 (till May 20th) regarding PPARs clinical applications in humans. The number of publications for subsequent years was extrapolated (dashed area).

Since 1992, new works have appeared steadily, following an increasing trend. At the peak of 2011–2012, up to 5 to 6 new works appeared daily. This rate is now lower. In 2023, they came in at about 3 per day (Figure 3). In light of the above, it is astonishing that, for example, typing “PPARs and 1960” into a search engine yielded as many as 9 hits instead of the expected zero, though none related to the year entered.

### 3.2. Electronic Search Strategy

Data for publication were collected from the Ovid MEDLINE, Embase.com, National Library of Medicine PubMed, and Web of Science databases. Predefined eligibility criteria excluded studies involving animal models. Peer-reviewed journal articles published from 1961 to 20 May 2024 were searched, limited to each year. A detailed search strategy was developed by best practices, as shown in Table 2.

Phrases were searched for in the title, abstract, and keywords provided by the author using Boolean Logic (‘OR’ and “AND”). Records retrieved in the Embase.com search were limited to only those absent from MEDLINE. The search filter was also adapted to retrieve studies involving human models. No restrictions were placed on language.

Discoveries about PPARs were searched using the terms: “pharmacological PPAR targeting”, “PPAR discoveries”, “medical PPAR investigations”, “peroxisome proliferator-activated receptor”, “PPAR AND Human”, PPARs AND disease”.

Free-text searches for all PPARs (α, β/δ, and γ) and pan-PPAR activity were linked to the terms agonist, modulator, stimulator, and activator using a proximity search so that both terms (e.g., “PPAR” and “agonist”) had to occur within five words of each other.

Reference lists of included original studies and relevant review articles obtained from the systematic search were manually reviewed to look for papers scientifically important or interesting to the authors. When a paper was deemed potentially relevant after reviewing the title and abstract, the full text of the study was downloaded. The literature search successfully identified relevant studies, some of which (285 papers) were included in this full-text review. The selection of cited papers was strictly subjective.

### 3.3. Calendar with Commentary

In 1989, 10 new papers arrived. Of these, we were particularly interested in the publication of Liu et al. [23], in which the authors demonstrate that replacing brown marrow with yellow marrow takes place under the control of PPARγ.

The years 1990–1991 did not bring much work regarding the study of PPARs in humans. Animal models (mice, horses) were mainly studied. Anyway, it is worth noticing the paper of Issemann & Green [2], where authors activated PPARs with growth factors, and Alvares et al. [24], who identified the heat shock protein HSP70 as a new possible member of the PPARs superfamily.

The year 1992: in this year, only 10 papers were published, among which, in our opinion, the work of Maksymowich et al. [25] should be singled out. They detected in all members of the steroid receptor superfamily (which form heterodimers with PPARs) a common conserved motif (the DNA sequence), which gives us hope for the future generation of pan-agonist or pan-antagonist PPARs. Then Gottlichet et al. found that chimeric ligands also work [26].

The year 1993 was one in which 29 new papers appeared in the PubMed database, among which it is worth mentioning the following: The paper by Keller and Wahli [27], whose authors see the links between endocrinology and dietetics on the grounds of PPARs. The works of Keller et al. [28] and Issemann et al. [29] in which the role of 9-cis retinoic acid in the activation and regulation of PPARs was demonstrated, and it was established that the mechanism leads through the binding of the said ligand to RXR. The work of Bardot et al. [30], whose authors studied PPAR-RXR heterodimers and acyl CoA oxidase and cytochrome P450 IVA6. The work of Boie et al. [31], whose authors tested the activity of enantiomers of known ligands. The work of Motojima [32], in which the author describes the various functions of PPARs and their activation mechanism. In the paper by Sher et al. [33], the authors describe the results of molecular studies, including cloning. Also interesting is the work of Bass [34], in which the author considers whether the functions performed by PPARs are more general or more specialized, and Gibson [35], in which he considers the future of PPARs. However, we were most impressed by the paper by Chen et al. [36], in which the authors claim to have evidence that there are not three PPARs but as many as five. If their argument is widely accepted, we are in for another nomenclature revolution, in which case the problems arising from the withdrawal of one receptor (beta/delta) will turn out to be a trifle indeed.

In 1994, 33 new papers were published, of which we highlighted the work of Kainu et al. [37], who demonstrated the brain receptor PPARα. In 2009, Aleshin et al. [38] confirmed this in rat brains. In addition, they revealed the presence of other isoforms in the brain, as did the work of Lemberger et al. [39], which showed that stress hormones could be ligands of PPARs.

In 1995, 49 new papers appeared, of which the following caught our attention: The work of Wahli et al. [40], in which the authors extensively discuss the panorama of lipid metabolism and its relationship with PPARs. The work of Gustafsson [41] in which it was pointed out that the toxicity of ligands may be a result of their binding to the receptor. The work of Bocos et al. [42] was repeated the following year by Lemberger et al. [43] (co-authored with W. Wahli), in which the authors found that fatty acids alone can activate PPARs. The paper by Yu et al. [44] described a new activator, HETEs, and its unusual properties. The paper by Keller et al. [45] (co-authored by W. Wahli), whose authors detail the role of RXRs in PPARs activation. The work of Juge-Aubry (co-author Wahli) [46], which considers the effect of thyroid hormone receptors, and the work of Baes et al. [47], who studied not an agonist but a PPAR-alpha antagonist.

The year 1996 brought 66 interesting publications, of which we would like to mention the following: The papers by Formann et al. [48] and Wilson et al. [49] in which the authors describe ligands and activators of PPARs, and the paper by Gibson, in which the author tries to anticipate the future of PPARs. In the Aubert et al. [50] paper, the authors describe the first activator from a new family (thiazolidinediones). The paper by Patel et al. [51] is one in which the authors show that thiazolidinediones brilliantly activate PPARγ but, on the other hand, promote atherosclerosis. Then there is the paper in which Brun et al. [52] analyze the differences between the response to the same stimulus of different isoforms of the receptors studied.

In 1997, 100 new papers arrived, and here are the most important among them: The paper by Guan et al. [53] reporting the presence of PPAR receptors in the human ureter, potentially bringing PPARs into the orbit of urology. In addition to these is the work of Bernlohr et al. [54], describing extracellular proteins and lipid transporters; the work of Clarke and Jump [55] with a description of an independent novel mechanism of PPAR activation by polyunsaturated fatty acids (at this point, one can make a loose hypothesis that this could be one of the reasons for the beneficial effects of PPARs on human health); and the work of Rove [56] on retinoid X receptors.

The year 1998 enriched our knowledge about PPARs with the results of 156 works, the most important of which, in our opinion, worked on the role of PPARs in the functioning of blood vessel walls [57] and skeletal muscles [58] as well as the impact of PPARs on lipid metabolism at the receptor and gene expression levels [59,60,61,62,63]. This has expanded our knowledge about the role of PPARs in metabolic syndrome and the development of atherosclerosis in humans. The inhibition of cell growth and induction of apoptosis by PPAR agonists was first demonstrated in human breast cancer cells [64]. In the same year, Sorensen et al. leaned into the problem of precisely controlling the activity of circumstantial PPARs, which takes on great importance in light of the planned preclinical studies [65], and Goodman found three independent connections between retinoids and schizophrenia [66].

The year 1999 brought us 233 new publications to read, many of which concerned the effects of PPARγ agonists on the growth and differentiation of human cancer cells in cancer tissues (ureter, bladder, prostate, liposarcoma) or cell lines such as colon cancer cells, T-cell lymphoma, or breast cancer cell lines [67,68,69,70,71]. The human T-cell leukemia retrovirus tax protein is a repressor of nuclear receptor signaling [72]. A possible role of reduced PPARγ activation by 15-HETE in the development or progression of prostate cancer has been suspected [73].

In 2000, there were 339 new interesting papers to review and evaluate. Lazennec G. and Wahli W. et al. observed a protein kinase A-dependent enhancement of the activity of all PPARα, β, and γ isotypes on two distinct promoters. Moreover, PKA inhibitors repress the PPAR ligands’ effect, suggesting that these ligands act partly by recruiting the PKA pathway. These findings highlight the involvement of the PKA pathway in PPAR action and indicate that it is essential for regulating gene activation by PPAR ligands [74]. Kawahito and co-authors demonstrated that the synovial lining layer, fibroblasts, and endothelial cells show expression of PPARγ in patients with rheumatoid arthritis (RA) and suggested that PPARγ may be an essential immunoinflammatory mediator and its ligands, especially 15d-PGJ2, may be helpful in the treatment of RA [75]. Much attention has been paid to the role of PPARs in oncogenesis, including PPARδ in human endometrial adenocarcinoma and colorectal cancer [76,77,78] and PPARγ in gastric or prostate cancer [79,80].

In exciting publications, Combs et al. showed that PPARγ agonists inhibit the β-amyloid-stimulated expression of the cytokine genes interleukin-6 and tumor necrosis factor α, which brings us closer to the mechanisms of Alzheimer’s disease [81]. In contrast, Zhou et al. drew attention to the over-representation of PPARγ sequence variants in sporadic cases of glioblastoma multiforme [82].

The year 2001 meant 437 new papers for us. Among them, Debrik et al. [83] found that PPAR-gamma has a pleiotropic function in the cell, while Chinetti et al. [84] detected functions performed by PPARs in blood vessel walls, which have serious health implications. Delerive et al. described PPARs in the context of modulation of the inflammatory response, which may have potential therapeutic applications in chronic inflammatory diseases [85]. The authors reported that PPARs activators participate in anti-inflammatory activation in various cell types by inhibiting the expression of pro-inflammatory genes such as cytokines, metalloproteases, and acute phase proteins. In addition, PPARs play an important role in negatively regulating the transcription of inflammatory response genes by antagonizing AP-1, nuclear factor-kappaB (NF-kappaB), a signal transducer and activator of transcription, and the nuclear factor signaling pathways of activated T cells. Duez et al. emphasize that PPARs play an essential role in modulating the development of atherosclerosis through actions at both the metabolic and vascular levels. PPAR-alpha and PPAR-gamma seem to be of the most significant importance in developing this disease, with high levels of their expression. Moreover, PPAR-alpha and PPAR-gamma are involved in immunoregulation, vasculitis, and thrombosis associated with atherosclerosis [86]. Also, Elangbam et al. presented that PPAR-alpha and PPAR-gamma receptors have a beneficial effect on inflammatory diseases, including atherosclerosis, by regulating the production of cytokines, the expression of adhesion molecules on endothelial cells, fibrinolysis, and the modulation of monocyte-derived macrophages [87].

2002: After reading 596 new papers, it can be concluded that, for example, in the review by Stumvoll and Haring, we can learn that the occurrence of the PPAR-gamma2—Pro12Ala polymorphism has a significant impact on the risk of common type 2 diabetes (T2D). The authors emphasized that a thorough understanding of the mechanism of this disease may allow for the prevention or improvement of T2D treatment [88]. Roberts et al. demonstrated that p38 MAP kinase activation is mediated by PPARα transcription and detectable changes in survival and proliferation. Moreover, they presented the problem of how MAP kinases interact with nuclear receptors such as PPARα. This process causes coordinate suppression of apoptosis and cell cycle progression [89]. On the other hand, Berger and Moller presented condensed knowledge about the relationship of PPARs to chronic diseases [90]. According to them, the strong therapeutic effects of PPARα and PPARγ agonists benefit systemic lipid levels, glucose homeostasis, and atherosclerosis. It was emphasized that PPARα activation can effectively alleviate dyslipidemia and that PPARγ agonists reduce insulin resistance. However, in the case of PPARδ, the authors suggest that it may also be an important therapeutic target for selected patients’ disorders, including cancer, infertility, and dyslipidemia. Fauconet et al. conducted studies in two different human bladder cancer cell lines, RT4 (derived from a stage I tumor) and T24 (derived from a stage III tumor). VEGF (mRNA and protein) is deferentially increased by the three PPAR isotypes. Additionally, the authors reported that only the MEK inhibitor PD98059 downregulated PPAR ligand-induced VEGF expression [91]. Moreover, Haydon et al. [92] showed that PPARγ agonists are excellent therapy for human osteosarcoma, the biggest metastasis problem in many late carcinomas, while Koumanis et al. [93] looked at correlations between overweight groups and mortality in association with PPAR G2 Pro12Ala genotypes. In contrast, Berger & Wagner [94] described both the health-promoting and pathogenic properties of PPARs.

In the following year, 2003, out of a group of 711, we considered the following to be particularly important. One is an article in which Francis et al. conducted clinical trials in which it was shown that only PPARα agonists improved the prognosis of atherosclerotic heart disease [95]. Another noteworthy work concerns PPAR-gamma ligands and their role as modulators in future cancer treatment strategies. Margeli et al. demonstrated the importance of PPAR-gamma in cell proliferation and cancer, establishing their anticancer properties against a wide range of cancer cells [96]. The following paper outlines the problem of using PPAR-gamma agonists thiazolidinediones (TZDs) to treat type 2 diabetes. Despite the use of TZDs and their ability to reduce and slow down the progression of insulin resistance, including lowering plasma glucose levels and significantly reducing triglycerides and free fatty acids in plasma, patients experienced increased body fat. However, it is suggested that fat redistribution takes place in a favorable direction, from visceral to subcutaneous stores [97].

In 2004, 959 fresh scientific reports were published. One of them is an article by Jiang et al. that pointed out that PPARγ may play an important role as one of the regulators of prostate cancer (PCa) cell differentiation and proliferation. Therefore, PPARγ agonists may find use as adjuncts to watchful waiting in selected subpopulations of patients with localized PCa [98].

In 2005, the number of new works exceeded 1000 when 1297 new papers were published. A big part of them seemed to be useful because they focused on the problem of the targets. For example, Bedu et al. [99] (co-author W. Wahli) described PPARs β/δ as a therapeutic target, and Tenenbaum et al. [100] used a ligand (H-LDL) to achieve a pan-PPAR effect in therapy. Buzzetti et al. examined the association of the PPAR-gamma2 Pro12Ala polymorphism with measurements of insulin sensitivity in a population of obese Italian children (mean age 10.38 +/− 2.8 years). The authors showed that the X12Ala variant (Pro12Ala or Ala12Ala) is significantly associated with greater insulin sensitivity in childhood obesity, which may, therefore, be protected against cardiovascular disease, obesity, and diabetes due to the phenotypic effect of the Ala 12 allele on insulin resistance [101].

The year 2005 also brought several studies of PPARs-specific ligands. A fascinating review paper by Tyagi et al. [102] describes the importance of homocysteine in endocardial function, structure, and remodeling in conjunction with nitric oxide metabolism in diabetic patients with impaired myocyte diastolic relaxation. According to the authors, homocysteine antagonizes PPARγ receptors, increasing oxidative stress and decreasing endothelial nitric oxide levels. Then nitrotyrosine content and matrix metalloproteinase activity in the diabetic heart increase, resulting in myocyte-endothelium disconnection. Harris et al. found that PPAR-gamma activation reduces head and neck cancer proliferation through the mechanism of action of 4-hydroxyphenylretinamide (4-HPR, fenretidine) [103]. In turn, Lowe et al. investigated oxazole-substituted indanylacetic acids in the character of PPAR-gamma and alpha activator ligand [104]. At the same time, LoVerme et al. showed that palmitoylethanolamide (PEA) is an activator of PPAR-alpha [105].

In 2006, the increase in the literature was even more significant, with 1343 new papers, of which Michalik et al. [106] (co-author W. Wahli) was selected. They wrote a valuable, well-done review in which the authors describe the selective effect of PPAR in vivo, which results from the interaction at a given time point between the expression levels of each of the three PPAR and RXR isotypes, the affinity for a specific PPRE promoter, and the availability of ligands and cofactors. Aung et al. investigated the role of peroxisome proliferator-activated receptors (PPARs) alpha and beta in differentiating HT-29 colon cancer cells and MCF-7 breast cancer cells [107]. The authors concluded that PPAR expression depends on the differentiation method and time. These studies have shown that changes in PPAR-alpha levels are not required to differentiate colon cancer cell lines, but changes in PPAR-beta are more closely related to differentiation. Trivedi et al. presented a different role of PPARs, showing that PPARs regulate sebum production. Moreover, this study’s authors believe that selectively modulating PPARs activity may constitute a new therapeutic strategy in treating acne [108]. Burdick et al.’s review consolidates information on the role of PPAR-beta in epithelial tissues. It presents critical discrepancies and how to direct research to fully understand how this receptor modulates epithelial homeostasis [109].

In 2007, there were 1577 new publications, some of which have vigorously stirred our imagination, among them a paper by Michalik and Wahli [110] on the differences of PPARs in healthy and diseased (acne, psoriasis, warts, and benign tumors) skin. Akhmetov et al. [111] claim to have found a link between the athletic performance of athletes and several PPAR polymorphisms. It is also worth mentioning the paper of Piqueras et al. [112]; they induced endothelial cell proliferation and angiogenesis using the activation of PPARβ/δ by various experimental ligands. In Grarup et al. [113], the authors report that in a sample of approximately 7500 white, elderly individuals, 12 polymorphic variants of the PPARβ/δ gene correlated with insulin resistance but not with diabetes type. Conversely, Hollingshead et al. conducted studies on human cancer cell lines (HT29, HCT116, LS-174T, HepG2, and HuH7) cultured in the presence or absence of serum and compared in vitro analysis with in vivo analysis. Based on the results obtained (the PPAR-beta/delta ligand did not increase cell growth or Akt phosphorylation, nor did it increase the expression of VEGF or COX2 in any cell line), the authors of this study concluded that PPAR-beta/delta ligands do not enhance tumor formation [114].

The year 2008 brought 1633 news items, and research about the relationship between PPARs and lung cancer is hidden among them. In one such study, Pedchenko et al. demonstrated that PPAR-beta/delta is highly expressed in most lung cancers. Furthermore, PPAR activation induces a proliferative response and survival in non-small cell lung cancer [115]. Borland et al. demonstrated that PPAR-beta/delta ligand activation inhibits keratinocyte proliferation through a PPAR-beta/delta-independent mechanism. Instead, retinoic acid’s inhibition of keratinocyte cell proliferation is mediated through a source of PPAR-beta/delta, contradicting the theory that retinoic acid would enhance cell proliferation through activation of PPAR-beta/delta [116]. One interesting study was conducted by Ahmed et al., who examined the expression of PPAR-beta in terminal ovaries and different histological grades of ovarian tumors. The authors of this study found that, unlike other cancers such as colon cancer, endometrial cancer, and head cancer, overexpression of PPAR-beta does not occur in ovarian tumors [117]. In the same year, Billin wrote a review paper in which he summarized the general knowledge available at that time about PPAR-beta/delta agonists and properties in the treatment of dyslipidemia and type 2 diabetes, as well as emerging clinical data on various PPAR-beta/delta agonists. Autor stated that at that time, their use in the treatment of dyslipidemia or type 2 diabetes could not be justified [118]. On the other hand, Gallardo-Soler et al. demonstrated that PPAR-γ/δ-mediated uptake of modified lipoproteins by monocytes, linking lipid metabolism and immunity, is an essential early event in the development of atherosclerosis [119]. In all cells involved in the atherosclerotic process, PPARs can modulate immune pathways through at least three different mechanisms: by directly binding to PPREs of anti-inflammatory cytokine genes, by transrepression of transcription factors such as NF-κB and AP-1; or by corepression [120]. Interestingly, in vitro studies demonstrated similar changes in AngII-treated macrophages: PPARδ activation increased total and free Bcl-6 levels and inhibited AngII activation of MAP kinases, p38, and ERK1/2. These studies uncover crucial proinflammatory mechanisms of AngII and highlight actions of PPARδ activation to inhibit AngII signaling, which is atheroprotective [121].

In 2009, out of 1666 new papers, we decided to give the following list of outstanding, groundbreaking work: Cavalieri et al. [122] tried to look at the role of PPARs (alpha) from the point of view of nutrigenomics, which brought the discussion closer to epigenetics. A theoretical review paper by Porcuna et al. [123] was finally published in 2021, in which the authors point out the great potential of epigenetic tools. In contrast, our first practical work in this field dates back to 2022. These studies demonstrated that the efficiency of PPARγ was increased in adipocytes obtained from insulin and isolated dogwood extracts. These results confirm the involvement of PPARγ in the regulation of cellular metabolism [124]. An interesting study was carried out by Danesi et al., in which green tea extract was added to the cardiomyocyte medium from the first seeding, as a result of which this extract selectively activated the PPAR-beta/delta isoform. Thus, the authors of this study observed that with the increase accompanied by the activation of PPAR-beta/delta, there was a decrease in the production of nitric oxide (NO) synthase and an increase in total antioxidant activity. Therefore, it has been suggested that the activation of PPAR-beta/delta may play a key role in reducing NO production [125]. It is worth looking at two publications of the same year on the role of PPAR-beta in oxidative stress-induced apoptosis in human umbilical vein endothelial cells (HUVEC). In Jiang’s publication, it was shown that H_2_O_2_ decreased the expression and activation of PPAR-beta, which played an essential role in H_2_O_2_-induced apoptosis in HUVEC [126]. A few months later, the same authors reported that repeated low-level H_2_O_2_ stress protected HUVECs from subsequent oxidative stress-induced apoptosis by increasing PPARβ expression and activity [127]. In turn, Bishop-Bailey and Bystrom summarized the current knowledge about the critical role PPAR-beta/delta plays in regulating inflammatory processes and immunity [128]. Ramanan et al. conducted research on patients after whole-brain irradiation (WBI). The authors demonstrated that PPARα ligands inhibited or slowed down radiation-induced pro-inflammatory responses in microglia in vitro. Moreover, they prevented the harmful effects of WBI on hippocampal neurogenesis in vivo, and the PPARγ ligand Pio alleviated WBI-induced cognitive impairment [129].

In 2010, the scope of knowledge expanded with the results of 1741 new works, including the work of Genovese et al. [130], who tested propenoic acid derivatives as PPAR-beta/delta agonist ligands. Grimaldi described the regulatory functions of peroxisome proliferator-activated receptor beta in terms of metabolism, inflammation, and cellular stress [131]. In his review, the author emphasized that activation of PPARβ using its strong and specific agonists prevented and reversed abnormalities associated with the metabolic syndrome. Therefore, both genomic and non-genomic modes of action can modulate responses to metabolic, inflammatory, and oxidative stress in several tissues. An interesting study was conducted by McKinnon et al., and it was performed on patients with endometriosis. The study showed that the expression of PPARγ in peritoneal endometriotic lesions was correlated with pelvic pain, dysmenorrhea, and dyspareunia experienced by patients [132]. This year also brought an exciting paper by Mistry and Cresci, who studied PPARs alpha, beta, and gamma to individualize heart failure therapy [133]. It is also important to mention the work of Bassaganya-Riera et al., who studied the effect of PPAR-gamma on respiratory infections with viruses, which offers potential hope for rescue in case of failed viral vector therapies [134].

In 2011, 1672 papers were published, mainly on the role of PPARs in lipid metabolism, inflammation, cell proliferation, and apoptosis. Clinically significant papers were precious. The role of T-cell PPARγ in lymphopenia-associated autoimmunity was determined for the first time. Housley et al. examined the role of PPARγ in CD4+ T cells and found that PPARγ expression in CD4+ CD25− T cells (Teff) is required to develop autoimmunity under lymphopenia. However, an unexpected function for PPARγ in Teff, in contrast to the documented immunosuppressive role of PPARγ, was found in increased Teff proliferation and survival [135]. A clinically valuable study of rheumatoid arthritis patients treated with pioglitazone showed a significant reduction in serum oxidative and inflammatory parameters and a significant improvement in DAS28 compared to the placebo group [136]. In turn, Sertznig and Reichrath presented an exciting summary of the effects of PPAR agonists in dermatology. This review summarizes the current knowledge of PPAR functions in various skin disorders, particularly inflammation and epidermal hyperproliferation (i.e., psoriasis, atopic dermatitis, acne, scleroderma, skin malignancies) [137].

In 2012, 1845 relatively fresh results arrived; the selected works are described below. First, Laschke and Menger [138] had developed a strategy for the use of PPARs in antiangiogenic therapy of endometriosis, while Nickkho-Amiry et al. addressed the modulation of PPARs, which reduces the growth of endometrial cancer cells [139]. Moreover, Knapp et al. [140] showed higher expression of PPARα and PPARβ and lower expression of PPARγ in patients with endometrial cancer than in patients with healthy endometrium. Additionally, Montero et al. [141] demonstrated that activation of PPARα and PPARγ modulates the expression of the human equilibrium nucleoside transporter hENT1, which plays a vital role in chemotherapy. Then Abbas et al. [142] found that a PPARγ agonist can both reduce and increase the risk of cardiovascular events, while Chen et al. [143] studied PPARγ receptors in neurodegenerative diseases, finding them to have a significant effect through NF-kappa beta. Additionally, Greene et al. [144] proved that the level of PPARδ increased during intense strength training and was negatively correlated with the concentration of total cholesterol and LDL, especially in the untrained state. In contrast, the level of PPARα increased intensively after exercise training.

Wadosky and Willis [145] showed in their paper that post-translational regulation of PPARs undergoes ubiquitination and sumoylation, and Peyrou et al. have dealt with PPARs in liver disease and found that they are essential in metabolic dysregulation [146]. Costa and Ciccodicola, on the other hand, investigated whether PPARγ is essential to diabetic retinopathy, finding, however, a beneficial role [147]. Then Balakumar and Mahadevan [148] argued in their paper that PPARs may be involved in the action of statins by showing anti-inflammatory, antioxidant, and antifibrotic effects. Wahli and Michalik [149], on the other hand, consider PPARs to be the main regulator of whole body metabolism. Furthermore, Reichenbach et al. [150] showed that the PPARα agonist Wy14643 inhibits cathepsin B protein expression in endothelial cells. Then, Taiileux et al. [151] claimed that PPARs have potential clinical applications in non-alcoholic fatty liver disease, as evidenced by their participation in modulating triglyceride accumulation. Vidella and Petinelli also mentioned the role of PPARs in NAFLD [152]. In this work, the authors showed that the reduction of PPARα levels is associated with the depletion of the n-3 polyunsaturated fatty acid chain, which may play a role in increasing the DNA binding capacity of the pro-inflammatory factors NFκB and AP-1, thus constituting one of the main mechanisms of progression of steatosis to steatohepatitis. PPARs also play a role in neurological diseases, as demonstrated by Bedendusi et al. [153]. The authors of this study proved that the progression of amyotrophic lateral sclerosis (ALS) leads to the activation of PPARγ in motor neurons, which contributes to the increase in the activity of lipid detoxifying enzymes, such as lipoprotein lipase and glutathione S-transferase α-2. PPARs may participate in the regulation of viral expression, as evidenced by the publication of Hu et al. [154] regarding the HBV virus. This experiment showed that PPARα regulates HBV gene expression through interactions with regulatory elements of the HBV promoter. Interestingly, Zuo et al. demonstrated that the induction of heat injury in fibroblasts activates the action of PPARβ, which has a protective effect on the structure and proliferation of these cells [155].

The same was true in 2013, with 1845 new papers, including the selected ones, such as the paper by Le Foll et al. [156], which analyzed the potential use of peroxisome proliferator-activated receptor (PPAR) agonists as promising new drugs in the treatment of psychoactive substance dependence based on preclinical studies. Aleshin and Reiser [157] described the roles of all PPAR isoforms. They addressed the regulation of oxygen signaling in the brain through pan-PPARs. The paper of Contreras et al. describes PPAR-alpha in the context of the key to metabolic and environmental adaptations of disease substrates [158], and the paper of Akyurek et al. describes PPAR-gamma in overweight children. These researchers found that PPAR-gamma levels in obese children were low [159].

In 2014 we can find 1688 research papers focused on PPAR, some showing the importance of PPARs in anticancer treatment. One such study was conducted by Yao et al. on human breast cancer cell lines (MDA-MB-231—estrogen receptor-negative and MCF7—estrogen receptor positive), where they showed that ligand activation and/or overexpression of PPARβ/δ inhibits the relative carcinogenicity of breast cancer and provides further support for the development of PPARβ/δ ligands to specifically inhibit breast carcinogenesis [160]. In addition, research into the treatment of HCV virus has been carried out. Researchers have shown that using fluoxetine, an HCV titer-lowering drug, also increases the activity of the peroxisome proliferator-activated receptor (PPAR) response element in HCV infection [161].

In 2015—1742 papers were published. One was Montagner et al.‘s paper, which reviews peroxisome proliferator-activated receptor PPARβ/δ function in skin wound healing and cancer. The work highlights the significant role of PPARβ/δ in inhibiting keratinocyte apoptosis at the wound edges through activation of the PI3K/PKBα/Akt1 pathway and its role during re-epithelialization in regulating keratinocyte adhesion and migration. The observations and analyses suggest the need to evaluate modulators of PPARβ/δ that attenuate or enhance its activity, depending on the therapeutic target [162].

In 2016, 1643 works were published. An exciting publication concerns the use of nanoparticles to enhance the anti-inflammatory potential of one of the PPARγ agonists—15-deoxy-Δ12,14-prostaglandin J2 (15d-PGJ2). Using solid lipid nanoparticles (SLN) and 15d-PGJ2 at low concentrations, 15d-PGJ2-SLN was developed and investigated for its immunomodulatory potential. In three inflammation models, 15d-PGJ2-SLN reduced neutrophil migration, increased IL-10 levels, and reduced IL-1β and IL-17 in peritoneal fluid. Therefore, using SLN may improve the therapeutic properties of the 15d-PGJ2 [163]. Also, in 2016, Xue et al. constructed the peptide-functionalized nanoparticle platform to deliver PPARγ activator rosiglitazone to adipose tissue vasculature [164]. This system promotes the transformation of white adipose tissue into brown-like adipose tissue and angiogenesis, which facilitates the homing of targeted NPs to adipose angiogenic vessels, thereby amplifying their delivery. In a diet-induced obese mouse model, compared with the control group, these angiogenesis-targeted NPs inhibited body weight gain and modulated several serological markers, including lipids and insulin. These findings suggest that angiogenesis-targeting moieties with angiogenic stimulator-loaded NPs could be incorporated into the clinical treatment of obesity and other metabolic diseases. A different type of carrier, i.e., diamond nanoparticles (DN) was used by Strojna et al. concerning the delivery of curcumin to cells [165]. The major problem with curcumin is its poor bioavailability, which can be improved by adding carriers. DN are large surfaces that are non-toxic, have antiangiogenic properties, and create bio-complexes through a fast and straightforward process of self-organization. The authors investigated the cytotoxicity of complexes of curcumin with DN against liver cancer cells and normal fibroblasts. Preliminary results confirmed the applicability of DN as an efficient carrier of curcumin, which improves its performance against cancer cells in vitro.

In 2017, 1606 papers were published. This year, Fidoamoere et al. [166] elucidated the role of PPAR-alpha in metabolic alterations of the oxygen-deficient microenvironment in newly formed tumors. The same problem was revisited 6 years later by Rolver et al. [167], who looked at the issue of creating, with the help of PPAR-alpha, an acidic microenvironment that favors young tumors. In the same year, Kado et al. [168] proposed to reduce ovarian cancer progression using the exact mechanism. Wright et al. researched human oral cancer cell lines CA-9-22 and NA, which were treated with the PPAR activators eicosatetraenoic acid (ETYA), 15-deoxy-δ-12,14-prostaglandin J2 (PG-J2), thiazolidinedione, and ciglitazone, and then tested on their ability to functionally activate PPARγ luciferase reporter gene constructs. The authors found a significant reduction in cell proliferation and clonogenic potential and concluded that with treatment, there was a decrease in cell proliferation and clonogenic potential [169]. A fascinating paper by Ivanova et al. describes the activation of PPARs in fatty acid and energy metabolism, stating that PPAR-gamma activation is related to atherosclerosis, cardiovascular disorders, and prognosis of cardiovascular surgery [170]. Gross et al. reported that PPAR ligands reduce comorbid disease by affecting WAT combustion storage capacity and fat combustion in BAT and/or peripheral possessions, thereby reducing ectopic fat load. Moreover, a well-prepared clinical study showed that the use of PPARγ has a beneficial effect in patients with non-alcoholic steatomyelitis [171].

The year 2018 brought us 1608 research papers. For example, Borland et al. showed that PPARs have a significant impact on carcinogenicity in squamous cell carcinoma [172]. The authors presented their results on the A431 human squamous cell carcinoma cell line, which showed that stable expression and activation of PPARβ/δ or PPARγ led to reduced carcinogenicity. Furthermore, Leiguez et al. found that in macrophages, the compound MT-III, snake venom phospholipase A2, activated PPARγ and PPARβ/δ and increased protein levels of both transcription factors and CD36 [173]. According to studies conducted by Sun et al. on the human hepatocellular carcinoma cell line HepG2, it is suggested that using 2,4-dichlorophenoxyacetic acid interferes with glucose metabolism in the liver by activating PPARβ [174]. Therefore, it leads to hepatocyte glycogen accumulation by increasing glucose uptake and improving gluconeogenesis (via FoxO1 and CREB).

2019 was particularly abundant in new (1509) and especially in critical reports, of which we have selected a few for presentation. One of the exciting and valuable publications is a review in which the authors present the issues related to chemotherapy-induced neuropathy and the effects of PPARγ activation in treating chemotherapy-induced neuropathic pain [175]. Vallée, et al. demonstrated that non-steroidal anti-inflammatory drugs acting as PPARγ agonists help regulate the WNT/β-catenin pathway and thus control tumor growth through cell cycle arrest, cell differentiation, and apoptosis and may reduce inflammation, oxidative stress, proliferation, invasion, and cell migration [176].

The year 2020 brought 1460 new papers. We found many publications dated that year on the links between PPARs and various types of cancer. One of them is an interesting work of Toraih et al. [177], who addressed the relationship between genotype and clinical prognosis of thyroid cancer patients, finding in a material analysis of 174 samples that the simultaneous presence of micro RNA 27a (miRNA 27a) and PPAR-alpha and RXR-alpha dimer are associated with adverse clinical prognosis. Hirao-Suzuki et al. demonstrated in the MDA-MB-23 human breast cancer cell model that COX-2 expression is positively modulated by PPARβ/δ-mediated signaling [178]. Moreover, Elie-Caille et al., using RTqPCR and Western blot analyses, showed that the peroxisome proliferator-activated receptor β/δ (PPARβ/δ) agonist GW501516 significantly reduced the expression of N-cadherin in the transitional cell carcinoma of the urinary epithelium of the bladder [179]. Moosavi et al. reported that in studies of Cao2 colorectal adenocarcinoma cells, extracellular vehicles EVs derived from gut microbiota such as *F. prausnitzii* increase the permeability of the intestinal barrier, among others, through the PPARα, PPARγ and PPAR β/δ genes [180]. Additionally, Liu et al. demonstrated that genetic variants in PPAR pathway genes, particularly MED1, PRKCA, and PRKCB, may contribute to susceptibility to pancreatic cancer [181]. Wouters et al. demonstrated that inflammation-induced PPARγ expression promotes myelin-induced foam cell formation in macrophages in multiple sclerosis [182]. PPARs are also associated with liver disease. Xia et al. showed that bergenine may be a promising drug candidate for treating liver fibrosis because it activated PPARγ, inhibited TGF-β, and reduced liver fibrosis by inhibiting hepatocyte necrosis and extracellular matrix formation [183]. In studies conducted in hepatocytes treated with non-esterified fatty acids, Shen et al. showed that choline and methionine regulate the transcriptional activity of PPARα and liver X receptor α (LXR-α) through phosphorylation of AMPK-α and regulate SREBP-1c independently of AMPK-α to promote lipid oxidation and transport [184]. Yu et al. showed that PPARβ/δ activating ligands increase the in vitro expression of decidualization biomarkers of endometrial stromal cells (ESCs), and PPARβ/δ antagonists impair decidualization markers [185]. In addition, Zhang et al. showed that the expression of PPARβ/δ was significantly increased in cholesteatoma, and the ligand-activated PPARβ/δ promoted the proliferation of cholesteatoma keratinocytes as a result of the positive regulation of the PDK1/PTEN/AKT/GSK3β/cyclin D1 pathway [186]. In the same year, Wójtowicz et al. [187] proposed using the PPARs in treating neurodegenerative disorders. More Manickam et al. [188] studied the effect of microbiota on skeletal muscle status, da Cruz et al. [189] attempted to control inflammatory processes through PPARβ/δ and manipulation of it with diet, and Wagner and Wagner [190] studied PPARβ/δ as a hallmark of cancer. In this news-rich year, additional mention should be made of Phua et al. [191] work (co-author W. Wahli), in which a PPARβ/δ agonist reduces inflammation in skeletal muscle. Additionally, Zhang et al. researched new biomarkers of myocardial damage. In this work, they demonstrated that small molecules transform myocardial energy metabolism by regulating and translating PPARα [192]. Interestingly, Andrade-Souza et al. researched the effects of exercise on nuclear transcription factors. Studies have shown that PPARα and PPARβ/δ gene transcriptions were increased when exercise was performed twice a day compared to once a day [193]. Furthermore, Faulkner et al. showed that in the human endothelial cell line HUVEC, the tubulogenic effects of compound GW0742 were dependent on PPARβ/δ and demonstrated a regulatory role for PPARβ/δ in regulating endothelial cell behavior and promoting tissue maintenance and repair [194]. In addition, Chai et al. showed that microRNA-9-5p inhibits extracellular matrix deposition and proliferation and induces apoptosis by targeting PPARβ in human hypertrophic scar fibroblasts [195]. It also turns out that PPARs have a potential role in treating diabetic retinopathy, as discovered by Capozzi et al. [196]. In this study, the authors showed that the PPARβ/δ inhibitor—GSK0660 inhibits the palmitic acid-stimulated production of inflammatory mediators by Müller cells. However, Contreras-Lopez et al. demonstrated a different property of PPARs. In their studies, the authors showed that PPARβ/δ participates in the regulation of the immunoregulatory potential of mesenchymal stem cells, therefore dictating their metabolic reprogramming and paving the way to enhance the immunoregulatory properties of MSCs and counteract their versatility [197]. Also of note from this year is the work of Petr et al., who studied the effect of genetic variants of PPARs on the performance of athletes [198]. In addition, Tutunchi et al. [199] reviewed the effects of oleoylethanolamide, a PPARα ligand, as a weight control agent.

There were 1404 newcomers in 2021, among which the following papers should be mentioned: Grabacka et al. [200] found that PPAR-alpha plays a key role in the processes of the innate immune response, and Willems et al. [201] highlighted the possibility of modifying PPAR-alpha (and also beta/delta) activity with photohormones. Kharbanda et al. [202] synthesized forty-eight molecules derived from the arylpropionic acid scaffold and evaluated them for their effects on diabetes based on the excellent docking point demonstrated by all structures made to the PPAR-γ receptor site. The authors concluded that arylpropionic acid derivatives may provide a new perspective toward developing antidiabetic agents with fewer side effects.

Several interesting review papers were also published in 2021. Christofides et al. [203] described the role of PPARs in immune responses, Rayner et al. [204] described PPAR-gamma-mediated neuronal regeneration, and Stark et al., in turn, described the role of PPAR-gamma in allergic diseases [205]. This year also saw the work of Hasegawa et al. who looked at liver diseases and described several potential new drugs using PPARs [206].

2022 was one of the peak years for publications on PPARs, with about 1555 of them recorded in the NCBI database. In our opinion, the most noteworthy are the papers regarding the relationships between non-coding RNA and PPARs. Previously, few works on this topic were published, but in 2022, according to NCBI, there were 146 of them. Therefore, review works are precious, especially those that concern common diseases whose pathogenesis is very complex and whose therapeutic goals are challenging to achieve. Such diseases include NAFLD, the most prevalent form of chronic liver disease in the world and a significant risk factor for developing hepatocellular carcinoma, and NASH. Non-coding, such as microRNA (miRNA) and long non-coding RNA (lncRNA), has been proven to play a significant role in the pathogenesis of these disorders. PPARs were found to be one of the major regulators in the progression of NAFLD. Mukherjee et al. presented reliable knowledge about regulating PPARs through ncRNAs and their role in NAFLD [207]. In the review on the clinical relevance of circulating non-coding RNAs in metabolic diseases (such as obesity, diabetes, cardiovascular diseases, and metabolic syndrome), Dandare cites that miRNA-138 suppresses adipogenic differentiation and is implicated in obesity by interaction with a potent inhibitor of adipogenic differentiation that interferes with Src homology region 2-containing protein (SHP2), an endogenous enhancer of adipogenic PPARγ [208]. A fascinating paper concerns miRNA networks, which form networks of systems and gene expression circuits through molecular signaling and cellular interactions and contribute to various health disorders [209]. It appears necessary to establish genomic signatures to determine multifactorial correlations and reveal variability observed in therapeutic effects, e.g., in patients with cardiovascular disease. Clinically validated miRNA biomarkers and relevant SNPs identified in clinical practice can serve this purpose. They should increase the ability to stratify patients, help select innovative therapeutic regimens, and identify innovative drugs and delivery systems. This paper examines miRNA–gene networks and SNP-derived genomic signatures to highlight specific gene signaling circuits as sources of molecular knowledge relevant to cardiovascular diseases.

The year 2023 resulted in 1310 new publications. We think the following should be mentioned: Kaltdorf et al. [210] proposed a new approach to evaluating experiments using PPARs and a new informatics tool called Boolean networks. Tanaka et al. reported [211] on PPARα and its ligand Ror 1, Ibrahim et al. [212] on the activation of SIRT 1 by taurine, and Yang et al. [213] on the effect of ligand PFAS (polyfluorolkyl) on the PPARα/ACOX 1 heteroduplex. Zhou et al. [214] described the impact of the FNDC5/PARGα heterodimer on macrophage pyroptosis. Nevertheless, in our ranking, the champions of 2023 were Pascoa et al. [215], presenting their prototype tool for identifying new ligands detected in cyanobacteria.

In 2024 (data from January to May 20, includes 385 papers), Kim et al. [216] elucidated the mechanism of PPAR-alpha’s effect on the intestinal stem cell pool in the process of calcification of blood vessels, while Liu et al. [217] tackled pectolinargenine, a Chinese folk medicine drug, demonstrating its multiple beneficial effects in many disease entities, including ligands and with the Nrf 2 gene. Exciting research was conducted by Cheng et al. [218], in which they led the study of the role of epigenetic regulation, including DNA methyltransferase 3a (DNMT3a)-mediated methylation and PPARγ-activated receptor inhibition, in the development of intervertebral disc degeneration (IVDD). The authors of this study showed that DNMT3a, by modifying the hypermethylation of the PPARγ promoter, activates the NF-κB pathway and thus contributes to apoptosis and degradation of the extracellular matrix.

In reviewing the literature, a trend in research has been noted: Chinese researchers are often concerned with green tea and drugs used in folk traditional Chinese medicine [219,220,221,222,223,224,225,226]. Indians, on the other hand, are keen on traditional medicines used in Ayurveda (mainly curcumin) [227,228,229]. Europeans, on the other hand, often try to demonstrate the beneficial effects of cannabis [230,231,232,233,234,235].

Examining this phenomenon in light of the statements about the lack of conflict of interest might be worthwhile.

## 4. Discussion

In a situation with so much interest in PPARs and such a huge scattering of detailed research topics, it is tough to summarize the results to date, let alone predict the future. Nevertheless, some things can be expected with reasonable probability. First, in pure science, it is possible to foresee a slow filling of the small gaps in knowledge that still exist. Second, in the field of pharmacology, there will be an acceleration of work on the search for new and better ligands, with little harm to human health. It is also most likely that pan-type approaches will become more common. In conclusion, the field is slowly moving from research to implementation, which bodes well for patients.

PPARα is a crucial regulator of lipid metabolism. It was discovered as the first member of the peroxisome proliferator family [236]. PPARα is mainly expressed in the liver and tissues with increased mitochondrial oxidation and fatty acid catabolism, such as brown adipocytes, cardiac muscle, skeletal muscle, and kidney [237]. PPARα activation leads to the upregulation of enzymes involved in fatty acid uptake, transport to mitochondria, and subsequent oxidation. This stimulates the breakdown of fatty acids, especially during fasting or prolonged exercise when glucose availability is limited and energy is needed. In addition, PPARα activation enhances the removal of circulating triglycerides in the blood by increasing the expression of lipoprotein lipase and other lipolytic enzymes, thereby lowering plasma lipid levels.

PPARγ is expressed in adipose tissue, the colon, the immune system, and the retina. Alternative splicing results in seven different mRNA transcript variants named PPARγ1 to PPARγ7 [238]. PPARγ influences the development, body localization, and adipose tissue metabolism [239]. In addition, it is a significant insulin sensitizer and regulates metabolism and cellular sensitivity to glucose and insulin. Moreover, PPARγ plays a vital role in cell differentiation and the regulation of apoptosis. Another function of PPARγ is to inhibit inflammatory processes, have an anti-atherosclerotic effect, and generally improve cardiac performance. PPARγ also plays an important role in human procreation (in women, it stimulates ovulation, while in men, it regulates sperm biology). When activated by specific ligands, PPARγ binds to the RXR receptor to form a heterodimer, and together, they regulate the expression of multiple genes [15,239]. The same can be observed in PPARα. Ligands for PPARγ can be both natural compounds (docosahexaenoic acid and eicosapentaenoic acid or other polyunsaturated fatty acids and some monounsaturated fatty acids such as oleic acid) and synthetic compounds (thiazolidinediones such as troglitazone, rosiglitazone, and pioglitazone) [240]. Non-steroidal anti-inflammatory drugs are also included in the PPARγ agonist group.

Numerous studies published to date point to the critical role of DNA methylation in regulating the expression and normal function of PPARγ in health and homeostasis by regulating many important vital processes [241,242,243]. PPARγ is also believed to play a massive role in the pathogenesis of many diseases in which dysregulation of DNA methylation leading to impaired expression disrupts the normal function of the transcription factor. Such disorders include insulin resistance and type 2 diabetes, and the epigenetic component plays a significant role in their development. Epigenetics are those changes in gene function inherited by mitotic or meiotic cells, which are not associated with changes in DNA sequence [244]. Such changes can increase or decrease the expression of a target gene [245]. DNA methylation profiles are greatly influenced by environmental factors. They also affect histone modification, leading to dysregulation of the expression of many genes, including the insulin signaling gene and lipid metabolism genes. In addition to metabolic disorders, epigenetics has been linked to cancer and neurodegenerative diseases [244].

PPARγ and PPARα homologs are subject to epigenetic regulation, mainly DNA methylation [123]. The PPARα promoter is hypermethylated, mainly in patients with non-alcoholic fatty liver disease (NAFLD) and patients with type 2 diabetes [246,247]. PPARα undergoes hydroxymethylation, which affects PPARα expression, predisposing a person to NAFLD [248]. Patients with metabolic syndrome have been found to have significant hyperlipidemia dependent on a different DNA methylation of PPARα [249]. Castellano-Castillo et al. demonstrated hypermethylation of multiple genes that correlated with metabolic dysregulation, including genes essential for regulating adipogenesis, lipid metabolism, and inflammation. This suggests that DNA methylation of the nuclear receptors PPARγ and PPARα is correlated with metabolic deregulation, the pathogenesis of metabolic syndrome, and the induction of inflammation [250].

Considering diabetes treatment, it is known that the use of an adjuvant PPAR agonist influences the control of glycemia, the reduction of HbA1c levels, and the improvement of insulin resistance compared to metformin monotherapy. Moreover, beneficial changes were observed in patients using PPAR agonists, such as a reduction in blood pressure and a decrease in the concentration of inflammatory markers and dyslipidemia. It was mentioned above that in the treatment of diabetes, PPARy agonists can be used, mainly thiazolidinediones (TZD), whose task is to prevent the effects of diabetes by increasing patients’ sensitivity to insulin, leading to a reduction in the concentration of both glucose and insulin in the plasma. The most commonly used TZDs are rosiglitazone, pioglitazone, and lobeglitazone sulfate in combination with metformin and other drugs used to treat diabetes [251]. Despite the positive effects of rosiglizatone, such as lowering fasting plasma glucose (FPG) and postprandial serum glucose, lowering HbA1c, and lowering insulin and C-peptide levels, this drug has clinically significant side effects such as edema, anemia, and weight gain [252]. Additionally, an increased risk of cardiovascular disease (CVD) has been observed in patients with unstable heart failure (HF) [253]. In turn, pioglitazone has antidiabetic effects, improves HbA1c, and has a positive effect on serum lipids; however, increasing the duration of pioglitazone use resulted in an increased risk of bladder cancer [254]. Nevertheless, unlike other TZDs, it has been shown that lobeglitazone can be a therapeutic substance that combines the features of both effectiveness and safety [255]. No side or carcinogenic effects were observed in patients taking it, but its antidiabetic effects have been proven [256].

The effect of PPAR agonists in treating diabetes may depend on individual factors such as single nucleotide polymorphism. One of the most frequently studied and analyzed polymorphisms is Pro12Ala, in which there is a nucleotide change from CCA-to-GCA in the codon12 of exon B of the PPARG gene [257]. Multiple studies have shown that the occurrence of this polymorphism was associated with a lower risk of type 2 diabetes as well as improved insulin sensitivity in humans, suggesting that this amino acid change enhances the action of insulin [258,259]. Additionally, it was shown that the occurrence of the Pro12Ala polymorphism was associated with a decrease in triglyceride content in white adipose tissue, skeletal muscles, and the liver, which was the result of increased leptin expression and a decrease in the lipogenesis process, which led to increased insulin sensitivity [257]. The reduction in immune function may result from several mechanisms. As mentioned earlier, pioglitazone is used to reduce insulin sensitivity in diabetes. Numerous studies also show that patients with the Pro12Ala polymorphism showed a better therapeutic response than participants with the Pro12Pro genotype [257]. Additionally, many studies show that the Pro12Ala polymorphism significantly impacts changes in HbA1C, FPG, and TG levels in T2D patients treated with TZD4 [260].

Particular attention should be paid to the importance of pharmacogenetics, which will make individual treatment based on the patient’s genetic profile possible in the future.

PPARs, playing an essential role in regulating the inflammatory response, have a significant impact on the course of diseases such as rheumatoid arthritis [75,136,261,262,263] and inflammatory bowel diseases [264,265,266,267,268]. In the synovium of rheumatoid arthritis (RA) patients, the abnormal migration, proliferation, and activation of fibroblast-like synoviocytes (FLSs) is observed in the joints. In vitro studies demonstrated that the expression of PPAR-γ in the synoviocytes of RA patients and adjuvant arthritis (AA) in experimental animals was significantly lower than that of normal FLSs [263,267]. Synthetic non-fibrate PPARγ ligands (thiazolidinedione family) and fibrates (bezafibrate and fenofibrate), as well as natural PPAR agonists (unsaturated and nitrated fatty acids, eicosanoids), had various effects on the severity and course of RA. In the synoviocytes of RA patients, the induction of inflammatory cytokine mRNA expressions such as TNF-α and IL-1β was significantly inhibited by the 15d-PGJ2, natural PPARγ agonist [261,269]. Using solid lipid nanoparticles may enhance this anti-inflammatory effect of 15d-PGJ2 and rosiglitazone [163]. A recent study has shown that PPARγ alleviates the inflammatory response in TNF-α-induced FLSs by binding to p53 in RA patients [270]. Another study indicated that PPARγ may induce the activation of Wnt/β-catenin signaling during FLSs activation [176,263]; perhaps in this way, PPARγ ligands induce synovial cell apoptosis.

Study results support the use of PPARγ agonists in the treatment of RA. Their beneficial effects, especially in combination with methotrexate, are documented. The mechanisms of this action include the inhibition of ROS and inflammatory cytokines generation, as well as the attenuation of the migration and proliferation of FLSs.

PPARγ is highly expressed in the colon (in epithelial cells and lamina propria mononuclear cells such as macrophages as well as T and B cells), being a key regulatory factor of bacteria-induced mucosal inflammation. A bacteria-induced excess of TLR4 under the influence of lipopolysaccharide (LPS) triggers the NFκB and mitogen-activated kinases (MAPKs) pathways, leading to increased production of inflammatory mediators. In patients diagnosed with inflammatory bowel disease (IBD); ulcerative colitis (UC), or Crohn’s disease (CD), as well as in experimentally induced colitis, the depressed PPARγ expression in colon epithelial cells has been shown. Simultaneously, the PPARγ gene has been described as a susceptibility gene for IBD [265]. One of the oldest anti-inflammatory agents used for the treatment of IBD is 5-ASA, the functional synthetic ligand for PPARγ in the colon. PPARγ is the critical receptor mediating the 5-ASA activity by trans-repressing several essential target genes such as NFκB, signal transducers, and activators of transcription [264]. Synthetic (thiazolidinediones, glitazars, non-steroidal anti-inflammatory drugs, aspirin) and natural (conjugated linoleic acid, barley leaf (BL), inosine) PPARγ ligands were used in various models of IBD, with different effectiveness [264,265,266,267,268]. Rosiglitazone was also used in patients suffering from UC, and the endoscopic and clinical results were promising [271,272]. As UC is closely associated with gut microbiota dysbiosis, Li et al., using 16S rRNA gene-based microbiota analysis, found that dietary supplementation of BL ameliorated dextran sulfate sodium (DSS)-induced gut microbiota dysbiosis and protects against DSS-induced colitis. The mechanism of this protective BL action resulted from improved intestinal mucosal barrier functions via the activation of PPARγ signaling. Similarly to the effect of exogenous treatment with inosine, BL protects against DSS-induced colitis by improving adenosine 2A receptor/PPARγ-dependent mucosal barrier functions [268]. The conjugated linoleic acid (CLA) induced PPARγ and δ and repressed tumor necrosis factor α (TNF-α) expression and NFκB activation while inducing the immunoregulatory cytokine transforming growth factor β 1 (TGF-β1). Clinically, CLA also ameliorated DSS- and CD4+-induced colitis through a PPAR γ-dependent mechanism [273]. Additionally, PPARγ receptors are directly involved in the mechanism of action of mesalazine, which is primarily used and effective in UC.

The impact of PPARγ on the course of IBD seems to pertain not solely to UC but also CD. Numerous animal studies have concluded that PPARγ agonists may have higher efficacy in maintaining rather than inducing IBD remission and that the therapeutic effect of PPARγ is mainly dependent on its abundance in target tissues.

It is also essential to emphasize the role of PPAR-alpha gene polymorphisms on cardiovascular risk.

The risk of coronary heart disease (CHD) has been suggested to be associated with polymorphisms of peroxisome proliferator-activated receptors. The results were controversial. In the case-control study, T allele carriers of C161T polymorphism were not significantly associated with CHD, while T allele carriers showed a higher risk of acute coronary syndrome (ACS). The meta-analysis indicated that compared with CC homozygous, T allele carriers had lower CHD but higher ACS risk. Other polymorphisms were also significantly associated with CHD risk under the dominant model: PPAR-alpha intron 7G/C polymorphism and L162V polymorphism [274]. The association of PPAR-alpha L162V polymorphism with cardiovascular risk may result from genetically conditioned changes in lipid metabolism. The serum total cholesterol and LDL-cholesterol levels were higher in PPAR-alpha V162 allele carriers in non-diabetic CHD. The increasing effect of the PPAR-alpha V162 allele on serum cholesterol levels was weakened with the presence of the PPAR-gamma 161T allele in the non-diabetic CHD patients. The ApoE4-PPAR-alpha V162 allelic combination of the ApoE/PPARA genes was found to be more frequent in diabetic CHD patients, independent of serum lipids. It was suggested that the PPAR-alpha L162V polymorphism may have diverse effects on serum lipids, and CHD risk depends on the presence of diabetes [275].

In our study, compared to healthy men, the frequency of the V allele of the L162V polymorphism was four times higher in men with confirmed coronary atherosclerosis. We concluded that L162V polymorphism in the gene for PPAR-alpha seems to be associated with atherosclerosis through a mechanism including regulation of the interleukin-6 level [276]. On the other hand, in patients with type 2 diabetes, there was a trend towards a lower prevalence of atherosclerosis and lower CHD prevalence in carriers versus noncarriers of the V allele. These data suggest that the PPAR-alpha polymorphism L162V might protect against the development of atherosclerosis or CHD in patients with DM-2 [277].

Recently, an increasing number of studies indicate that various PPAR gene polymorphisms influence the effects of PPAR agonists on lipids and cardiovascular risk. Moreover, much evidence shows that lipid metabolism is influenced by gene-gene and gene-environment interactions involving PPAR-alpha genes [278].

**Figure 4:**
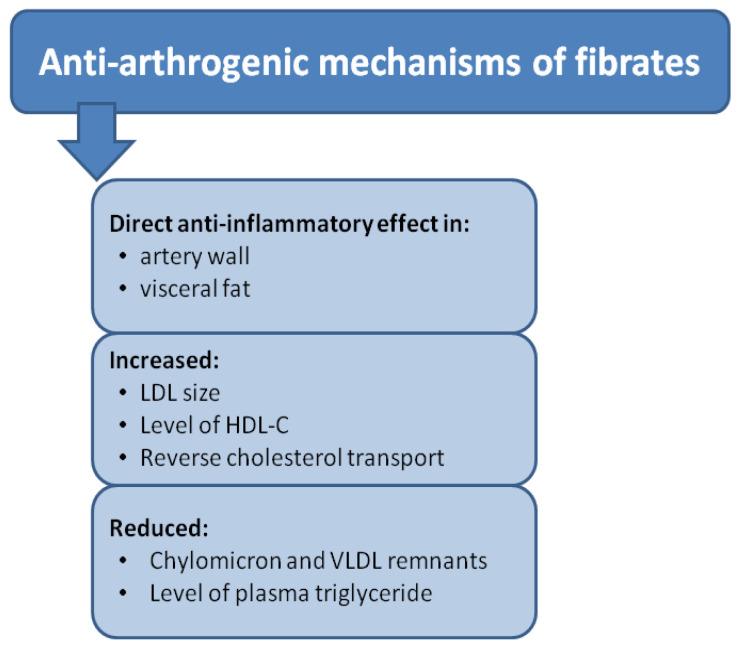
Anti-arthrogenic mechanism of fibrates.

The data presented above indicate that PPAR-alpha agonists, by reducing metabolic disorders associated with atherogenic dyslipidemia, reduce the so-called residual risk of cardiovascular events. Currently, scientific societies recommend the use of PPAR-alpha agonists in people with hypertriglyceridemia after the LDL cholesterol level has been reduced to the target values. An attempt to reduce the non-HDL cholesterol to the target values can be considered (a) combination therapy using omega-3 fatty acids (PUFA, at 2–4 g/day) and statin in patients with triglyceride concentrations above 2.3 mmol/L (200 mg/dL) despite statin treatment; (b) combination therapy using choline fenofibrate and statin can be considered as a part of primary prevention in patients with triglyceride concentrations above 2.3 mmol/L (200 mg/dL) whose LDL cholesterol levels have been reduced to the target values, especially where the HDL cholesterol levels are low; (c) combination therapy using choline fenofibrate and statin should be considered in high-risk patients with triglyceride concentrations above 2.3 mol/L (200 mg/dL) whose LDL cholesterol levels have been reduced to the target values, especially where the HDL cholesterol levels are low [279,280]. The anti-arthrogenic mechanism of fibrates is shown in Figure 4.

**Figure 5:**
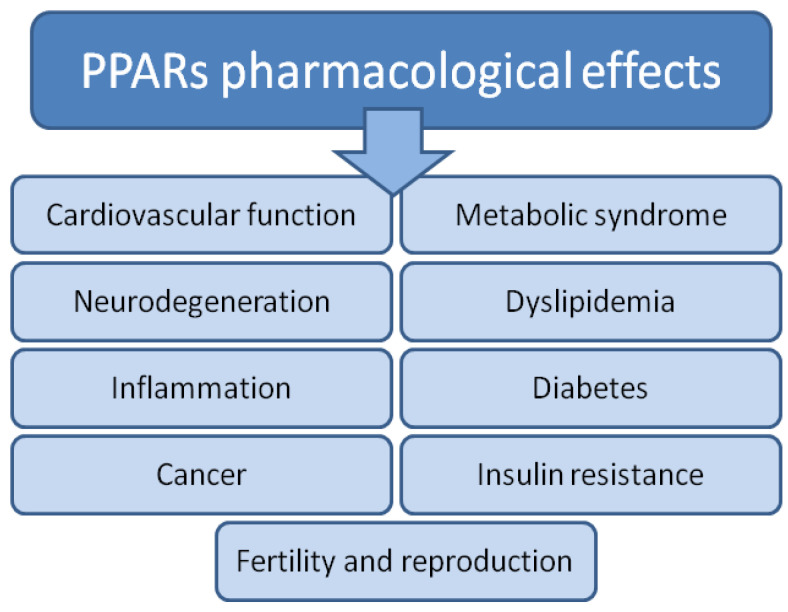
PPARs pharmacological effect.

PPAR agonists already have and may have many more medical applications now and in the future (Figure 5).

This problem was widely discussed by Cheng et al. in their fascinating review exploring PPAR modulators in medical research, showing their application and utility. The authors collected and described existing knowledge about different classes of PPAR agonists, which are under various phases of clinical investigations or have already been approved for medical use [281]. Anyway, it is also worth mentioning the paper by Colapitro et al. [282], which describes new therapeutic fibrates and PPAR agonists such as bezafibrate and fenofibrate. In addition, the paper describes other PPAR ligands, such as Seladerpar, Elafibranor, and Saroglitazar, tested in patients with PBC (Primary Biliary Cholangitis). Bezafibrate has the most promising effect on liver disease, improving symptoms in patients with PBC. However, despite its broad spectrum of activity, it should be used with caution in patients with chronic kidney disease and those with cirrhosis, in whom it increases bilirubin levels and impairs liver function. However, in patients who do not yet have cirrhosis it could be used successfully. Seladelpar is a PPAR-δ agonist. It has choleretic properties, resulting from altered bile acid synthesis, and anti-inflammatory properties. Elafibranor, a dual PPARα/δ agonist, is a promising drug due to the lack of adverse effects of PPAR-γ ligands on the heart. Saroglitazar, on the other hand, is mainly a PPAR-α activator and exerts agonist effects on PPAR-γ; that is, it has dual PPAR agonist activity. It is used to treat NASH, diabetic dyslipidemia, and diabetes uncontrolled by statins. Its effects are also being studied in PBC. These drugs show improvement in ALP but have only been tested in the short term and on a small group of patients. Further studies are needed to determine the dose and test the drug’s safety. Ideally, these patients should be selected for individual double or triple combination therapy.

Unfortunately, side effects are the main problem in designing and developing new synthetic PPAR ligands. Therefore, future PPAR research should focus on minimizing the side effects of PPAR agonists and the potential of PPAR antagonists. Using such compounds will open a new chapter in using PPAR ligands in medicine.

**Figure 6:**
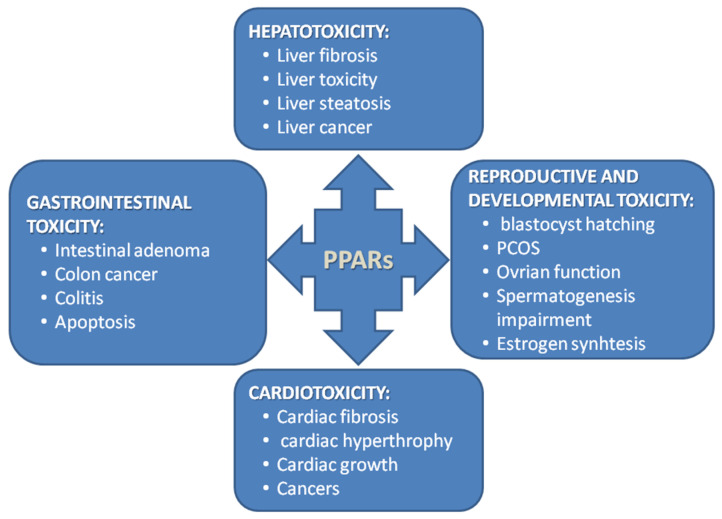
Systemic toxicities of PPARs.

Generally, ligands of PPARs can be agonists (full, partial, or inverse) or natural antagonists. There are 84 types of PPAR synthetic ligands used as drugs to treat various diseases. For example, PPARα agonists (fibrates), PPARγ agonists (thiazolidinediones -TZDs) and combined PPARα/γ agonists (glitazars) are therapeutic agents for CVD prevention, T2DM and dyslipidemia. Unfortunately, PPAR ligands are reported to have multiple organ toxicity (Figure 6) [283].

TZDs can cause hepatotoxicity, heart failure, or fluid retention. Glitazone’s side effects include body weight gain, peripheral edema, and congestive heart failure [283]. Fenofibrate therapy causes pulmonary embolism, pancreatitis, an evaluated plasma creatinine level (reversible after therapy ends), and an increased rate of noncardiovascular mortality. However, fibrate risk should be treated individually [284].

## 5. Concluding Remarks

For 35 years, knowledge about PPARs has significantly expanded. Over the years, thousands of studies have been performed. PPARs are an exciting group of transcription factors due to their connection with various chronic diseases, such as cancer, diabetes, obesity, arteriosclerosis, and neurodegenerative diseases. PPAR agonists are used to treat these diseases. For example, PPARα agonists treat hyperlipidemia or modulation of inflammatory response, while PPARγ agonists have potent hypoglycemic potential in patients with insulin resistance and cancer [285]. However, time and many studies are still needed to confirm the safety and effectiveness of the long-term use of PPAR agonists. In addition, particular attention should be paid to individual variations, such as polymorphisms that influence the function of PPARs. However, more research is still needed to fully understand the functions of PPARs.

## Figures and Tables

**Table 1:** Pan-PPAR effect of PPARs: when one type of receptor is activated, the rest is inhibited. ↑—means receptor activation, while ↓ means receptor inhibition.

Possibilities	PPARα	PPARβ/δ	PPARγ
1	↑ activity	↓ activity	↓ activity
2	↓ activity	↓ activity	↑ activity
3	↓ activity	↑ activity	↓ activity

**Table 2:** Data to be extracted from databases.

Category	Description
Study ID	Authors Year Journal
Model	Human
Intervention	PPAR isotypes: - PPARα - PPARβ/δ - PPARγ - pan-PPAR
Other	The term “PPAR” reported in the title or abstract—yes/no Criteria for inclusion and/or exclusion of data provided—yes/no
Search term type	Free-text

## Data Availability

Data are contained within the article.
